# Evaluation of lifestyle of female adolescents through latent class analysis approach

**DOI:** 10.1186/s12889-019-6488-8

**Published:** 2019-02-13

**Authors:** Valter Paulo Neves Miranda, Paulo Roberto dos Santos Amorim, Ronaldo Rocha Bastos, Vitor Gabriel Barra Souza, Eliane Rodrigues de Faria, Sylvia do Carmo Castro Franceschini, Silvia Eloiza Priore

**Affiliations:** 1 Programa de Pós-Graduação em Saúde Coletiva (UFJF), Laboratório de Informações Geo-referenciadas (UFJF), Juiz de Fora, Brazil; 2 Pós-Graduação Stricto Senso em Educação Física (PPGEFIUFV), Universidade Federal de Viçosa, Viçosa, Brazil; 3 Programa de Pós-Graduação em Saúde Coletiva (UFJF), Laboratório de Informações Geo-referenciadas (UFJF), Universidade Federal de Juiz de Fora, Juiz de Fora, Brazil; 4 Departamento de Nutrição, Universidade Federal de Juiz de Fora, Juiz de Fora, Brasil; 5 Programa de Pós-Graduação em Ciência da Nutrição, Universidade Federal de Viçosa, Viçosa, Brasil; 6 Programa de Pós-Graduação em Ciência da Nutrição, Viçosa, Brasil

**Keywords:** Lifestyle, Adolescents, Latent class analysis, Cluster analysis, Physical activity, Sedentary behavior

## Abstract

**Background:**

Lack of regular physical activity, high sedentary behavior and presence of unbalanced alimentary practices are attitudes associated with an inadequate lifestyle among female adolescents.

**Objective:**

to assess the lifestyle of female adolescents based on measurements of behavioral variables.

**Methods:**

Cross-sectional study with 405 female adolescents between 14 and 19 years old, resident and attending public schools in Viçosa (state of Minas Gerais). Their lifestyle was analyzed by the Physical Activity Recall, number of steps, screen time (ST), cellphone time (CT), sitting time, food frequency questionnaire (FFQ), and alcohol and tobacco consumption. With multiple correspondence analysis it was possible to observe dispersion and approximation of the variables’ categories. Latent class analysis (LCA) was used for modeling the “lifestyle” variable, having been conducted in the poLCA (Polychromous Variable Latent Class Analysis) package of the R statistical software.

**Results:**

The mean age was 15.92 ± 1.27 years. Most of the adolescents were considered physically inactive (78%) and with low number of steps (82.57%); 41.45% reported not performing Moderate to Vigorous Physical Activities (MVPA) adequately. Sedentary behavior was found high when assessing ST (72.90%) and CT (65.31%). It was found the best fitted latent class model for the lifestyle (p-G^2^ = 0.055, p-χ^2^ = 0.066) featured three latent classes and one covariate (alcohol): Class 1, ‘Inactive and Sedentary’ (γ = 77.5%); Class 2, ‘Inactive and Non-sedentary lifestyle (γ=16.31%); and Class 3, ‘Active and sedentary’ (γ=6.19%). Female adolescents that had ‘never consumed alcohol’ were 2.26 times as likely (log OR = 0.8174; *p* = 0.033) to belong to class 3 (Active & Sedentary lifestyle) than to class 1 (Inactive & Sedentary lifestyle).

**Conclusion:**

Latent class analysis model with five manifest variable (MVPA, number of steps, ST, sitting time and number of meals) and alcohol consumption like covariate showed itself to be an accurate and objective method in the assessment of female adolescents’ lifestyle. Female adolescents that had ‘never consumed alcohol’ were more as likely to belong to class ‘Active & Sedentary lifestyle’ than to class Inactive & Sedentary lifestyle. An inactive and sedentary lifestyle is coupled to other unhealthy behaviors during adolescence, possibly carrying over into adult life.

## Background

Adolescence is a transition phase from childhood to adult life in which growth and development occur at increasing speed [1]. Furthermore, identity development in educational and interpersonal domains becomes increasingly intertwined over time [2]. During that period, healthy or unhealthy habits may be acquired or consolidated, like physical inactivity and sedentary behavior, which will remain for the rest of one’s life.

Lack of regular physical activity, increase in sedentary behavior and presence of unrecommendable, unbalanced alimentary practices are examples of attitudes associated with an inadequate lifestyle among adolescents, due to the link with obesity, risk factors for cardiometabolic diseases and psychosocial problems [3–5]. Over half of all adolescents, in general, do not reach the minimum daily recommendation of 60 min of moderate and vigorous physical activities (MVPA) [6]. This prevalence is yet higher among female individuals in the late phase of adolescence, setting in which physical inactivity may reach 70% [7].

Generally speaking, adolescents may stay three thirds of their day in sedentary activities, i.e. those with energy expenditure short of 1.5 MET (metabolic equivalent) [8]. This sedentary behavior is also evaluated by the amount of time spent in front of devices with a screen (televisions, computers, tablets, smartphones and video game consoles). Physical inactivity and sedentarism may be associated with the increase in consumption of energetic, ultra-processed foods, rich in sugars and sodium, as well as with the decrease in ingestion of fruits, vegetables and dietary fibers [5].

When investigating the lifestyle of a specific population with heterogeneous characteristics, like adolescents, a person-centered approach can offer more insight than a variable-centered approach [4]. Person-centered techniques aim to group similar individuals based on their characteristics, focusing on relations among individuals rather than variables; regression models are typical of the latter [9]. Latent class analysis (LCA) is a subset of Structural Equal Modeling (SEM), a precise and judicious way of clustering that can be used in the assessment of lifestyle, based on the investigation of behaviors commonly adopted by adolescents [10].

The latent variable, or construct, is not measured directly, but rather in an indirect fashion through two or more variables. LCA does not impose a predefined concept of that which is being observed, thus it is an approach more focused on the individuals’ features, which may be homogeneous or heterogeneous depending on the actual data structure [11].

The evaluation of behaviors related to adolescents’ lifestyle in a single latent variable may show relations with other health outcomes. Especially for girls, who have shown themselves to be more inactive and sedentary than boys [12].

There is scarcity of studies in the literature which take behavioral and social variables jointly to predict the lifestyle of female adolescents, especially through the method of latent class analysis [10, 13]. Given the context above, the objective of this study is to assess the lifestyle of female adolescents through the measurement of behavioral manifest variables.

## Methods

This is a descriptive and analytic cross-sectional study.

### Sample

The study population consisted of female adolescents 14 to 19 years old, resident and attending public schools in Viçosa (state of Minas Gerais, Brazil).

A cluster sampling plan was used, proportionally to the number of adolescents enrolled in the selected public schools (clusters). A design effect, estimated at 1.1, was also introduced to correct the variance of parameter estimates, accounting for intra-cluster correlations. Public high schools were queried about the number of female students aged between 14 and 19; in 2014 there were 1657 students in this age range.

Based on that, the sample size was calculated in StatCalc from the EpiInfo software, version 7.2.0.1 (Georgia, United States). Sample size calculations considered a confidence level of 95%, maximum acceptable error of 5% and prevalence of 50% for outcomes [14] (e.g. physical inactivity level), and yielded a result of 344 students plus an additional 15% to allow for possible losses, adding up to a minimum of 396 students. To reach this minimum sample size, the two schools with the greatest amounts of eligible students were selected to make up the sample.

To ensure a validation of information that were collected of female adolescents, inclusion and the exclusion criteria were observed. The Inclusion criteria analyzed if the adolescents have been14 to 19 years old, have had undergone menarche at least one year earlier, have accepted voluntarily participation, have been good conditions of health, and have had authorization signed by the parents or legal guardians if under 18 years old. As exclusion criteria we have observed if the female adolescents could realize any kind physical activity normally, if they have had any chronic or communicable disease or some factor associated with obesity or inflammation diseases, if have been using some kind of controlled drug or medicine that could influence the functioning of metabolism, and being part of another study involving assessment of either body composition or nutritional status control. Both criteria were observed throughout the process of selection and data collection, from the contact with the students in the school, in the Health Division and in the research laboratory of the Federal University of Viçosa.

### Ethical considerations

The study was approved by Federal University of Viçosa’s Committee for Ethics in Research with Human Beings and filed on the Brazil Platform under the number 30752114.0.0000.5153, decision 700.976/2014. The present project followed the rules set forth by Brazilian National Health Council Resolution 466/12. Each volunteer only took part in the project after turning in the Assent Form and the Informed Consent Form, signed respectively by herself and by her parents or legal guardians. The Assent and Informed consent were written by all participants and participants parents/guardians in the case of minors. All procedures performed involving human participants were in accordance with the ethical standards of the institutional and/or national research committee and with the 1964 Helsinki declaration and its later amendments or comparable ethical standards.

### Data collection procedures

Data collection procedures started in June 2014 and finished in December 2015. The first stage took place in the schools, after consulting with and getting approval from the direction. The students then received an explanation about the procedures and were given the Assent and Informed Consent forms to be properly signed and handed back. Both contained detailed descriptions of the project and assured the safety, confidentiality and privacy of the information collected.

The second stage happened at the Health Division of the Federal University of Viçosa. Sociodemographic data and alcohol and tobacco use information were collected by members of the research project previously trained for their tasks.

Age was calculated in the *WHO AnthroPlus* software and categorized according to the World Health Organization classification (WHO) according to the phase of adolescence: intermediate, from 14 to 16 years, and late, from 17 to 19 years. Socioeconomic classification was based on the questionnaire proposed by the Brazilian Association of Survey Companies [15].

The last stage in the project was the assessment of lifestyle-related behavioral measures. The instruments for evaluating physical activity level and sedentary behavior, as well as the pedometer, were thoroughly explained at the Health Division and then handed over to the students. Completed questionnaires and pedometers were retrieved at the two participating schools.

### Lifestyle assessment

Lifestyle was assessed as a latent variable by a latent class model [16]. Physical activity level, sedentary behavior, food frequency, and alcohol and tobacco use were the behavioral variables used to make inferences about the latent variable. All behavioral manifest variables were measured during eight days.

The first day of data was discarded to minimize the *Hawthorne* effect, which consists of a change in one’s behavior to comply with what is supposedly expected by the study [17]. All measurements recorded were analyzed separately for weekdays and weekends.

The *Digiwalker* SW 200 (Yamax, Japan) pedometer measured the number of steps. The volunteers were instructed on proper usage of the device—which should be placed at the waistline on the right side of the body—and received a piece of paper with these instructions and a space to write down their daily number of steps. A researcher was continuously available at the schools during the data collection period to provide help in the event of questions or problems. The cutoff points proposed by Tudor-Locke et al. [18] classified the number of steps. The female adolescents were inactive when the mean count was lower than 11,700 steps per day.

The 24-h physical activity recall (24hPAR) assessed habitual physical activities, which were classified according to their specific metabolic equivalent for adolescents [19]. The energetic compendium was used to help with notes on what kind of physical activity was being done during the 8 days. In this instrument, participants wrote down the activities done over a 24-h period in intervals of 15 min. Activities were considered moderate to vigorous physical activities (MVPA) if they had a metabolic equivalent (MET) equal to or greater than 3. The MET corresponds to a multiple of a person’s basal metabolic rate, the power required to stay at rest, which is typically represented in the literature by an oxygen consumption of approximately 3.5 O_2_ mL/kg/minute.

MVPA was adequate when a minimum daily time of 60 min on average was practiced by female adolescents [20]. Physical activity level (PAL) was calculated as total energy expenditure divided by basal metabolic rate [21]. The values obtained were categorized according to the cutoff points proposed by the Institute of Medicine (IOM) [22].

Sedentary behavior was assessed by screen time (ST), cell phone screen time (CT) and sitting time during weekdays and weekends. ST and CT were measured according to the questionnaire proposed by Miranda et al. [23]. Students reported they spent too much time in front of a cell phone screen, often surpassing other screen devices. That being the case, it was decided that CT should be measured separately from the other devices. Both ST and CT were considered high when their means were greater than or equal to 120 min [24].

Section four of the *International Physical Activity Questionnaire* (IPAQ) [25] analyzed volunteers’ sitting time on weekdays and weekends. The weighted mean of these values was employed as an overall measure. Due to the lack of a specific cutoff point, the 75th percentile (75^th^P) was used for classification purposes; for the all-days average it was 585 min.

Food frequency was assessed by a simplified version of the Food Frequency Questionnaire (FFQ), only observing the number of times a week the food types were consumed. FFQ information was analyzed through the mean of the number of days in the week each food type had at least one of its members consumed.

FFQ classification was done using two-step cluster analysis (TSC) [26]. For each food type, individuals were classified as showing either adequate or inadequate food frequency, taking as reference the 75^th^P for that type. Specifically, “fruits” (75^th^P = 6), “vegetables” (75^th^P = 7), “tubers” (75^th^P = 4), “dairy products” (75^th^P = 7) and “cereals, bread and pasta” (75^th^P = 7) were considered inadequate when less than 75^th^P, and, conversely, “sugars and sweets” (75^th^P = 7), “oils and fats” (75^th^P = 7) and “condiments” (75^th^P = 7) were considered inadequate when equal to or greater than P75. TSC analysis identified three groups, labeled as healthy FFQ, moderately healthy FFQ and unhealthy FFQ.

The daily number of meals was computed from the responses to breakfast, second breakfast, lunch, afternoon tea, dinner and supper. The mean value for all seven days was calculated and subsequently categorized relative to the 50th percentile (50^th^P = 4.0). Values equal to or less than 50^th^P were considered a low number of meals.

Alcohol and tobacco use was observed by two modules of the short version of the G*lobal School-Based Student Health Survey* (GSHS). The first module is composed of five questions about ingestion of alcoholic beverages, while the second module comprises six questions involving cigarette smoking or other forms of exposition to tobacco. The GSHS was developed jointly by WHO and the Centers for Disease Control and Prevention, and later was translated into Portuguese and validated for use with Brazilian adolescents [27].

Answer options labeled “a” meant no sort of alcohol consumption or tobacco use in any situation and received a score of 0. Other answers were coded with increasing scores as they indicated greater exposure to such substances. Therefore, the sum of all answers being zero meant the adolescent had never used alcohol or tobacco.

In order to be considered valid and included in the analyses, all behavioral variables were measured for at least five weekdays and two weekend days. The overall, weekday and weekend means were calculated for the variables under consideration. After thorough verification of the data, values deemed inconsistent or invalid were excluded.

### Statistical analysis

The database was created in the Statistical Package for the Social Sciences (SPSS) for Windows, version 20.0 (IBM Corporation®, New York, 2016), with double data entry and verification. Statistical analyses were conducted with the help of SPSS and the *R* statistical software (R Development Core Team, 2014), version 3.2.2 (“Fire Safety”). A significance level of α = 5% was adopted throughout in interpreting test results.

Kolmogorov-Smirnov tests for normality and assessments of asymmetry and kurtosis were used to check whether the data followed normal distributions, and to select between parametric or nonparametric tests accordingly.

For a descriptive analysis of the variables, medians and interquartile ranges were calculated. The Mann-Whitney test was employed for comparative analyses between two independent groups, and the Wilcoxon signed-rank test compared weekdays and weekends within a same group for differences.

For categorization and clustering of food frequency information, two-step cluster analysis (TSC) was used [26]. The rules for choosing the number of groups were based on the best combination of low Bayesian Information Criterion and presence of more categories with importance values close to 1.

Multiple correspondence analysis (MCA) was performed as a preliminary step which verified correspondence, dispersion and approximation of the variables’ categories. With this exploratory method it was possible, through graphical representation of the principal plane, to have an idea about the indicator variables and the number of latent classes for the model. The distribution of the categories and their internal correlation coefficient were analyzed by the inertia value and Cronbach’s alpha for each dimension.

LCA was used for modeling the “lifestyle” variable. This method is more appropriate for analysis of interactions and associations between different kinds of behavioral variables. This method was selected because it is a person-centered approach, and hence can offer better conditions to evaluate heterogeneous and asymmetric variables, as those related to the lifestyle of adolescents [4, 9, 10]. Most of categorical indicator variables had cutoff points validated for Brazilian female adolescents.

LCA was conducted in the *poLCA* package (*Polytomous Variable Latent Class Analysis*) [16] available in the library of the *R* statistical software.

Firstly, ten manifest variables—PAL, habitual PA, expending energy total, MVPA, number of steps, ST, CT, sitting time, FFQ, number of meals—were chosen for being hypothetically linked to the adolescents’ lifestyle; then, age, socioeconomic class, tobacco and alcohol use were chosen for covariates.

Diagnostic evaluation of the most parsimonious model—that which offers the best description of manifest variable observations for the least parameters estimated (which depends on the number of manifest variables and covariates)—was done considering the Akaike Information Criterion (AIC), Bayesian Information Criterion (BIC), chi-squared goodness-of-fit test (χ^2^) and entropy. Model quality with the inclusion of covariates was evaluated by likelihood ratio tests (G^2^).

## Results

In total, 611 female adolescents were invited to participate in the project; however, 206 were not included, of whom 131 did not accept to participate and 75 either did not match inclusion criteria or did not properly complete some of the requested procedures. Of the 75 excluded girls, 10 reported having undergone menarche at least one previous year, 12 reported that they could not do physical activity because of medical recommendations, 17 were using recent contraceptive or some medication that could alter the functioning of metabolism, 16 did not deliver correct the terms of assent and concern and 10 participants declare that they are participating in another research project.

Therefore, 405 students went through the whole assessment, being 275 from the first public state school and 130 from the second. Mean age was 15.92 (±1.27) years, with 259 students (69%) in the intermediate phase and 146 (31%) in the final phase of adolescence. With regard to socioeconomic class, 64.7% belonged to classes B2 and C1, 21.2% to classes A1, A2 and B1, and 14.1% to classes C2 and D.

Most students were classified as having low PAL by the IOM (78%) and as being inactive by the pedometer (number of steps) (82.57%). 41.45% reported not performing at least 60 min of MVPA daily. Sedentary behavior was high, both as assessed by ST (72.90%) and by CT (65.31%) (Table 1).

**Table 1:** **–** Frequency analysis of the variables associated with female adolescents’ lifestyle. Viçosa-MG, Brazil, 2018

Categorical variables	Absolute frequency	Relative frequency (%)
Age (*n* = 405)		
Intermediate period	266	65.7
Late period	139	34.3
PAL (*n* = 392)^a^		
Sedentary	21	5.35
Low PAL	295	75.25
Active	72	18.36
Very Active	4	1.04
MVPA (*n* = 386)^a^		
Inadequate MVPA	160	41.45
Adequate MVPA	226	58.55
^b^Number of steps (*n* = 396)^a^		
Inactive	327	82.57
Active	69	17.43
ST (*n* = 369)^a^		
High ST	269	72.90
Adequate ST	99	27.10
CT (*n* = 369)^a^		
High CT	241	65.31
Adequate CT	128	34.39
cNumber of meals (*n* = 392)^a^		
Normal	174	44.4
Low	218	55.6
^d^FFQ (*n* = 396)^a^		
Healthy FFQ	147	37.2
Moderate FFQ	123	31.1
Unhealthy FFQ	127	31.7
Alcohol (*n* = 405)^a^		
Has consumed or consumes	74	56.30
Never consumed	177	43.70
Tobacco (*n* = 405)^a^		
Has used or still uses	253	62.50
Never used	152	37.50

*PAL* Physical Activity Level, *ST* Screen time, *CT* Cell phone time, *MVPA* moderate to vigorous physical activities, *FFQ* food frequency questionnaire

^a^Exact number of each variable. ^b^The cutoff value of the total number of steps was 11,700, according to Tudor-Luke et al. [18]

cThe food frequency classification was performed using the 50th percentile = 4 of the number of meals during a week

^d^*Two Step Cluster* Analysis classified the food frequency questionnaire. The quality of this analysis was average (0.5) and the between-groups proportion ratio was 1.19

With respect to the number of meals, it was seen that 218 (55.6%) of the girls had up to 4 meals a day. TSC showed that the best fitted model used three clusters, based on the information from the food frequency questionnaire (FFQ): healthy FFQ (37.2%), moderately healthy FFQ (31.1%) and unhealthy FFQ (31.7%).

Over 50% (56.3%) of the adolescents reported having tried or making ongoing use of alcoholic beverages; 62.4% confirmed they had been exposed to tobacco at some point in the past, or that they either had tried it or made ongoing use of it. The prevalence of alcohol use and tobacco exposure in the past 30 days was, respectively, 26.4 and 27.7%.

Positive correlation was observed between the physical activity measures (PAL and number of steps). These variables displayed negative correlation with screen time, which in turn had positive correlation with weekend sitting time (Table 2). Alcohol and tobacco use had positive correlation between them and with cell phone time and age.

**Table 2:** Correlation matrix between behavioral variables of female adolescents’ lifestyle. Viçosa-MG, Brazil, 2018

Variables^a^	PAL	MVPA	Number of step	ST	CT	IPAQ weekdays	IPAQ weekend	NM	Alcohol	Tobacco	Age
PAL	1	0.038	0.252^**^	−0.155	−0.064	− 0.099	−0.069	− 0.058	0.095	0.062	0,095
MVPA	–	1	0.009	−0.034	−0.006	0.042	0.037	0.020	−0.068	−0.035	− 0,021
Number of step	–	–	1	−0.056	−0.125^*^	− 0.051	−0.042	0.019	0.046	0.061	0,048
ST	–	–	–	1	0.008	0.062	0.17^*^	0.06	0,018	−0.45	−0,39
CT	–	–	–	–	1	0.118^*^	0.154	−0.062	0.162^*^	0.143^*^	−0,005
IPAQ weekdays	–	–	–	–	–	1	0.582^**^	−0.072	0.001	−0.008	−0,111^*^
IPAQ weekend	–	–	–	–	–	–	1	−0.008	−0.073	− 0.099	−0,07
NM	–	–	–	–	–	–	–	1	0.076	−0.048	0,116^*^
Alcohol	–	–	–	–	–	–	–	–	1	0.458^**^	0,105^*^
Tobacco	–	–	–	–	–	–	–	–	–	1	0,064

*PAL* Physical Activity Level, *MVPA* Moderate to Vigorous Physical Activities, *ST* Screen time, *CT* Cell phone time; *IPAQ* International Physical Activity Questionnaire, *NM* number of meals

^a^ Variables without normal distribution. *Spearman’s Correlation* “rs”. ^**^ *p* < 0.001; ^*^ *p* < 0.05

Comparison tests between behavioral variables, such as the Mann-Whitney and Kruskal-Wallis tests, evidenced the most physically active adolescents showed greater number of steps (*p* < 0.001) and shorter screen time (*p* = 0.05). On the other hand, those who informed greater use of alcohol (*p* = 0.003) and tobacco (*p* = 0.033) showed greater cell phone time.

It was also possible to see that students walked fewer steps (p < 0.001) and spent more screen time (*p* < 0.001) on weekends. During weekdays, though, they stayed longer in a sitting position (*p* = 0.020).

**Fig. 1:**
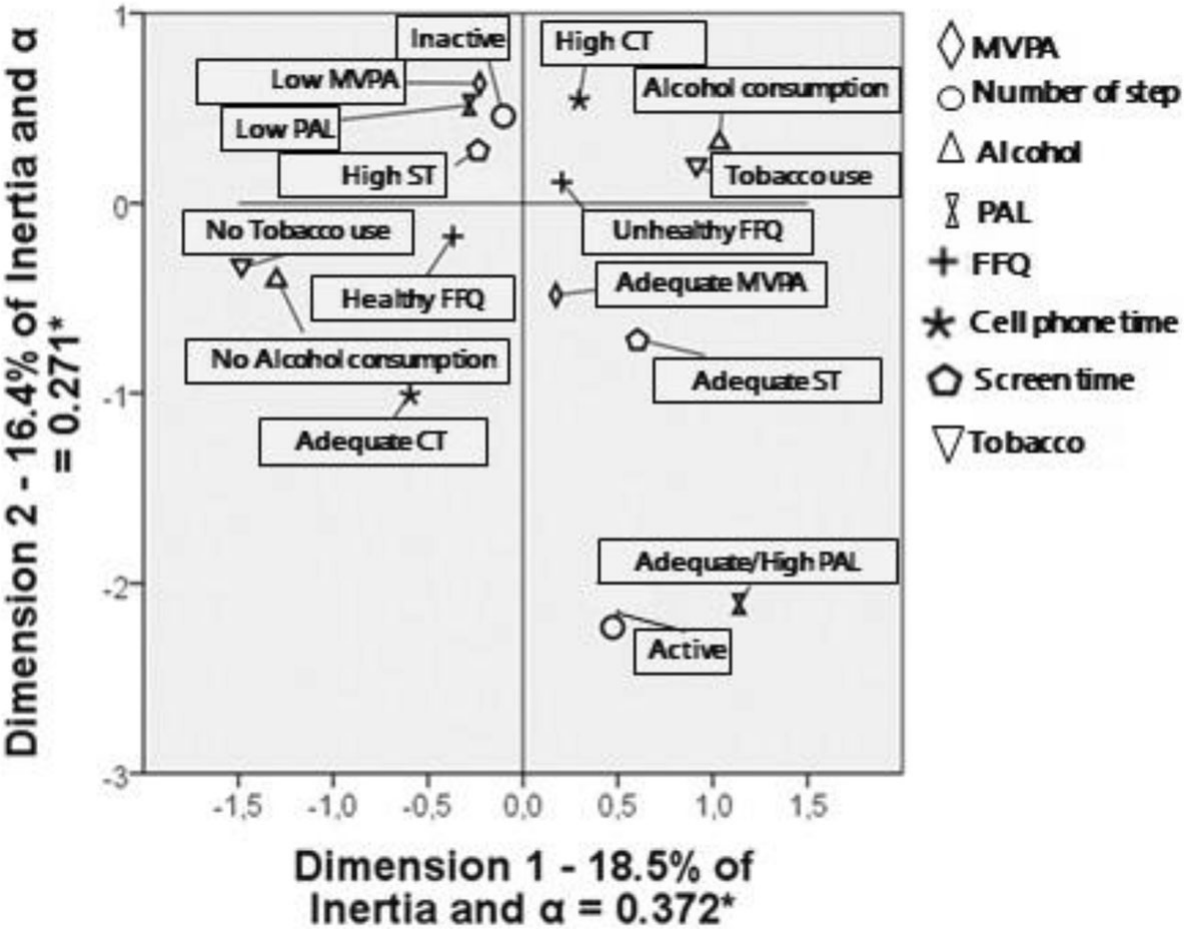
– Multiple Correspondence Analysis of variables related to female adolescents’ lifestyle. Viçosa-MG, Brazil, 2018. * Cronbach’s α. MVPA: Moderate to Vigorous Physical Activities; PAL: Physical Activity Level; FFQ: Food Frequency Questionnaire; ST: Screen time; CT: Cell phone time

MCA gave a graphical representation of the association between different measures of lifestyle. Dimensions 1 and 2 explained together 34.9% of the total variability; the internal correlation coefficients (Cronbach’s α) were 0.372 and 0.271, meaning moderate internal correlation (Fig. 1).

Responses related to a healthy lifestyle (Adequate/High PAL, adequate MVPA, active and adequate ST) were in the same quadrant. The same happened with responses characteristic of a less healthy lifestyle (low PAL, low MVPA, inactive and high ST). It was also possible to notice that high TC, unhealthy FFQ and alcohol and tobacco use were located close together.

When fitting the latent class model, the selection of manifest variables MVPA, number of steps, screen time, number of meals and total sitting time, coupled with the use of 3 latent classes, led to better values of fitness and parsimony. Specifically, this model achieved AIC = 1995.54; BIC = 2019.86; Pearson’s goodness-of-fit χ^2^ for multiway response profile table = 13.98 (d.f. = 14, *p*-value = 0.45). In comparison with other models using different manifest variables and numbers of latent classes, this model had overall superior metrics and also enabled better interpretability of classes, which warranted its selection as our basic, no-covariates latent class model (Table 3).

**Table 3:** Latent Class Analyses models of female adolescents’ lifestyle. Viçosa-MG, Brazil, 2018

Tem Manifest Variables: PAL, HPA, TEE, MVPA, Number of steps, ST, CT, Sitting time, FFQ, Number of meals								
Class	AIC	BIC	G^2^	χ^2^	Residual df	*p-*G^2^	*p*-χ^2^	Entropy
2	3666.532	3745.666	423.1982	562.6456	299	2.8813E-06	2.2832E-18	0.8568
3	3639.644	3760.231	374.3107	480.5879	288	0.0004561	7.7335E-12	0.8649
4	3638.019	3800.057	350.6859	455.4848	277	0.00176593	7.358E-11	0.9634
5	3647.572	3851.061	338.238	442.8939	266	0.00178086	5.5058E-11	0.9384
Five manifest variables: MVPA, Number of steps, ST, Sitting time, Number of meals								
Classes	AIC	BIC	G^2^	χ^2^	Residual df	*p*-G^2^	*p* -χ^2^	Entropy
2	1947.37	1988.992	20.575704	19.3121	20	0.4224731	0.5016259	0.3794
3^a^	1955.54	2019.865	16.745866	13.9849	14	0.2699738	0.4508331	0.5635
4	1958.315	2045.343	7.520696	7.3486	8	0.4816307	0.4995252	0.7188
5	1966.37	2076.101	3.575722	3.5636	2	0.1673177	0.1683342	0.6871
Covariates								
Tobacco	1951.419	2023.312	17.88298	17.9882	12	0.11929017	0.11604908	0.75166
^b^Alcohol	1952.332	2024.224	20.64676	20.0565	12	0.05579853	0.06602311	0.7935
Alcohol +Tobacco	1952.641	2032.102	18.66259	19.5953	10	0.04476354	0.0333205	0.5447
Age	1938.599	2010.492	30.40539	25.6985	12	0.00242546	0.01183853	0.9891
Age + Tobacco	1926.784	2006.244	24.72117	22.2802	10	0.00589984	0.01373898	0.8554
Age + Alcohol	1936.877	2016.338	27.47129	23.3220	10	0.00219242	0.00961824	0.8636
Age + Alcohol +Tobacco	1928.754	2015.782	25.11389	22.3723	8	0.00148695	0.00427082	0.8620

*PAL* physical activity level, *HPA* habitual physical activity, *MVPA* moderate to vigorous physical activities, *TEE* Total Energy Expenditure, *ST* screen time, *CT* cell phone time, *IPAQ* International Physical Activity Questionnaire, *FFQ* food frequency questionnaire, *AIC* Akaike Information Criterion, *BIC* Bayesian Information Criterion, *G*^*2*^ likelihood ratio, *p-G*^*2*^ likelihood ratio test**,** ***χ***^***2***^ Chi-squared, *p-* ***χ***^***2***^ Chi-squared test (*Goodness of fit)*

^a^LCA modeling fittest; ^b^The best model with alcohol like covariate

**Fig. 2:**
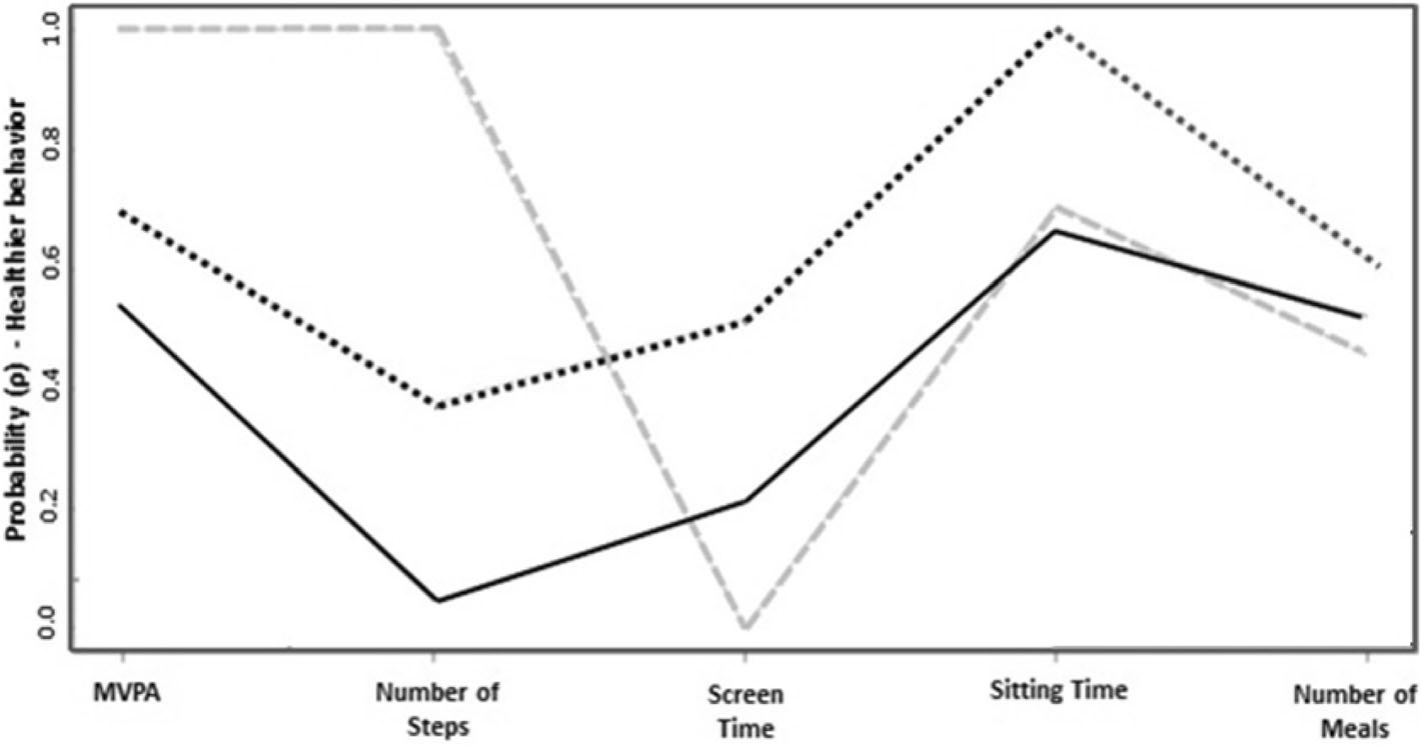
– Profile plot of the Latent Class Model of female adolescents’ lifestyle. Viçosa-MG, Brazil, 2018. *Prevalence (γ) of latent classes. ρ: item-response probability Class 1: Inactive & Sedentary Lifestyle – γ = 0.775; Class 2: Inactive & Non-Sedentary Lifestyle – γ = 0.1631; Class 3: Active & Sedentary Lifestyle – γ = 0.0615. MVPA: Moderate to Vigorous Physical Activities.

To account for the effect of covariates, that is to say, observed variables that have no intrinsic role in the definition of the latent variable, but nevertheless exert indirect influence on it, we added all possible combinations of available covariates to the model above. Alcohol use was the only covariate to be chosen because it allowed for far better interpretability, homogeneity and separation of item response patterns in the latent classes (Fig. 2), while it maintained a good model fit, with AIC = 1952.33, BIC = 2024.22, *χ*^2^ = 20.06 (d.f. = 12, *p*-value = 0.066) and entropy = 0.79; in fact, it provided a significant improvement in likelihood versus the no-covariates model, with G^2^ = 8.52 (d.f. = 2, p-value = 0.014).

After analysis and interpretation of the item response probabilities, the lifestyle classes were named as: Inactive & Sedentary lifestyle (class 1), with 252 observations (γ = 77.5%); Inactive & Non-sedentary lifestyle (class 2), with 52 observations (γ = 16.31%); and Active & Sedentary lifestyle (class 3), with 21 observations (γ = 6.15%) (Fig. 2).

Female adolescents classified as having an “Inactive & Sedentary” lifestyle have nearly 0% probability of reaching the recommended number of steps (average of 11,700 steps), about 20% probability of having normal screen time (less than 2 h/day), and over 50% probabilities of attaining recommended MVPA (minimum of 60 min), sitting time and number of meals.

Alcohol consumption was a predictor of membership in latent classes of female adolescents’ lifestyles (Table 4), especially differentiating between classes 3 (Active & Sedentary lifestyle) and 1 (Inactive & Sedentary lifestyle). Female adolescents that had ‘never consumed alcohol’ were 2.26 times as likely (log OR = 0.8174; *p* = 0.033) to belong to class 3 (Active & Sedentary lifestyle) than to class 1 (Inactive & Sedentary lifestyle). The association between class 2 and class 1 wasn’t significative (*p* = 0.781).

**Table 4:** Alcohol as a predictor of membership in latent classes of female adolescents’ lifestyle. Viçosa-MG, Brazil, 2018^a^

	Coefficient	SE	Odds Ratio	CI(95%)	t value	*p*
Class 2 (Inactive & Non-Sedentary lifestyle) / Class 1 (Inactive & Sedentary lifestyle)						
α (Intercept)	−1.171	0.721	0.310	0.075–1.275	−1.623	0.130
β (Alcohol)^b^	0.070	0.2470	1.072	0.661–1.740	0.284	0.781
Class 3 (Active & Sedentary lifestyle) / Class 1 (Inactive & Sedentary lifestyle)						
α (Intercept)	−2.86406	0.60536	0.05703	0.017–0.0186	−4.731	< 0.001^*^
β (Alcohol)^b^	0.81745	0.33908	2.264	1.165–4.401	2.411	0.033^*^

*CI95%* Confidence interval 95%, *SE* standard error

^a^Logistic regression analysis output by poLCA. ^b^never consumed. ^*^(*p* < 0.05)

## Discussion

Latent class analysis showed that the lifestyle of female adolescents can be assessed mainly by information relative to the practice of physical activity and to sedentary behavior. The best fitted model had five manifest variables (MVPA, number of steps, screen time, number of meals, total sitting time and number of meals) and three latent classes.

None of the identified classes contained individuals at the same time physically active and non-sedentary. Class 1, “Inactive & Sedentary” lifestyle, had the greatest prevalence (77.5%). Class 2 was labeled “Inactive & Non-Sedentary” lifestyle, with members having roughly 70% probability of being physically active as assessed by MVPA and number of steps. On the other hand, this class was the healthiest in terms of sedentary behavior: around 50.0% of girls did not have high ST and 100% displayed adequate sitting time.

Lastly, in class 3, “Active & Sedentary” lifestyle, the item response probabilities of manifest variables showed that 100% of female adolescents reached the recommendation of PA for both MVPA and number of steps. As for sedentary behavior, 0 and 70% were classified with adequate ST and sitting time, respectively.

Going beyond, it was possible to test the influence of alcohol consumption as a covariate. Logistic regression analysis showed that female adolescents that had never tried or consumed alcohol were more likely to belong to latent class 3, ‘Active & Sedentary’, than girls who declared having tried or drinking alcoholic beverages (OR = 2.26, *p* = 0.033).

Usually, others variables such as age, gender and socioeconomic status are used as covariates in latent class analyses of adolescents, as they may influence the practice of physical activity, sedentary behavior and eating behavior, according to some authors [10]. In the present study, neither gender nor socioeconomic level influenced the LCA model. Specifically, the variable gender was by design constant across all participants, who were female adolescents. The reason for restricting the study to girls was the result found in current research that female adolescents are more physically inactive and more sedentary than their male counterparts, especially after the early phase of adolescence [12].

Lawler et al. [28] also used latent class analysis and revealed six distinct classes for girls (Organized Run/Swim & Dance/Gym; Organized Dance; Leisure Active Team Sport; Active Individual Sport; Walk/Run/Outdoor games; Non-Participation). This same study also found that girls in team or individual sports and boys in team sports demonstrated significantly higher self-determined motivational characteristics than other physical activity profiles.

In our study there was overall greater prevalence of unhealthy behaviors, with the majority of girls being inactive and having high ST, high CT, low number of meals, high exposure to alcoholic beverages and early introduction to tobacco. An advantage of using LCA is that the multiple features that classify the individuals may be examined together, rather than separately, thus avoiding an increase in type I error [9]. Furthermore, according to Fitzpatrick et al. [29], LCA has advantages over more conventional analytic approaches as it does not limit the number of possible classes or groups and allows for assessment of interactions between different types of behavior [30].

The lifestyle classes generated in the present study were identified from different behavioral measures of the adolescents. Physical activity, sedentary behavior and number of meals constituted the relevant feature set (indicators) to construct the latent variable of interest.

Broad literature shows obesity to be the biggest public health issue across the world and to have been considerably increasing in the past years, thanks to the confluence of multiple lifestyle-related factors [3]. Childhood and adolescence are key periods of interest because they may potentially be a prime opportunity for the development of obesogenic behaviors, which can stabilize or further increase during adult life [31].

Physical inactivity is the top fourth cause of death in the whole world. Furthermore, together with sedentary behavior it can be associated with non-communicable diseases coronary diseases, like type 2 diabetes mellitus, breast and colon cancer [32]. In this study, ST and CT were considered excessive for 70% of the adolescents. It is well established that sedentary behavior may cause hazards to adults’ health [33], although that information is still not fully confirmed in childhood and adolescence [34].

Nevertheless, sedentarism at younger ages must be controlled since children and adolescents with this behavior high or altered have greater odds of becoming inactive and sedentary adults [35]. Children and adolescents are currently part of the digital generation and use devices, applications, video games and the Internet excessively at ever earlier ages [36].

Data and indicators from the survey carried out by the Brazilian Internet Steering Committee (CGI) and the Regional Center for Studies on the Development of the Information Society [37] evidenced that 23.7 million children and adolescents in Brazil, or 80% of their total, are Internet users. And for 83% of them the cell phone has become their main electronic device.

Assessment of food frequency revealed that around 60% of the adolescents do not eat fruits and vegetables on a regular basis, besides ingesting more often fats, sweets and sugars. It was also noticed that more than half of the students reported having equal to or fewer than 4 meals a day.

Philippi and Leme [38] investigated food frequencies of 1661 female adolescents in São Paulo (Brazil) and verified that ingestion of fruits, juices and vegetables was under recommended values in approximately 95% of the sample. The authors also observed that 66 and 93% of the subjects were above recommended levels, respectively, for oils and fats and for sugar and sweets eaten [39]. In addition to the low ingestion of fruits, vegetables and cereals, omission of breakfast and substitution of a snack for either lunch or dinner are observed among female adolescents [38].

In this current study, meal skipping was a very frequent habit among adolescents. According to Hyanos et al. [40], depressive symptoms and low self-esteem were significantly associated with the initiation of disordered restrictive eating. Furthermore, poor family communication/caring and maternal dieting significantly predicted long-term risk for escalating restrictive eating severity, whereas individual body image issues (i.e., weight concerns, body dissatisfaction) and social concerns (i.e., weight-related teasing, peer dieting) were significant short-term correlates of initiating disordered restrictive eating.

Another result worthy of mention was the considerable exposure of the adolescents to alcohol consumption and tobacco use. Over half of the girls have at least once made use of some type of alcoholic beverage or tobacco, and 25% reported frequent use of alcohol and cigarettes. Adolescence may be considered a period of greater vulnerability to the use of alcoholic beverages and products containing tobacco, since the individual is undergoing a process of physical, cognitive and mental development [41].

The consumption of alcoholic beverages by adolescents is worrisome because of both the greater tendency to impulsivity at this stage of life and the damage caused to brain development by alcohol. Adolescents’ use of alcohol, tobacco, marijuana, and other illicit drugs is a significant public health concern due to its prevalence and associated negative health and psychosocial consequences [31, 42].

Menezes et al. [43] observed that 8.9% of male and female adolescents reported having used at least one cigarette in the previous 30 days. Also in this study it was identified that the late phase of adolescence and the presence of parents or friends who smoke were the factors most strongly associated with the use of cigarettes. It has been seen that people starting smoking and drinking habits during adolescence may carry them over into adult life. From these relevant observations, it was decided our study should test alcohol and tobacco as covariates.

One shortcoming of this study was the dichotomous encoding of the behavioral variables used for LCA, which might underestimate the effect of a possible subcategory. It was necessary to employ it, though, and it also made construction and interpretation of the model easier.

Another aspect that deserves further consideration was the assessment of some measures of physical activity, sedentary behavior and food frequency by questionnaires or recalls. Some measures were adopted to improve the accuracy of analyses with these variables; for example, physical activity was examined both in a subjective and an objective way, with the number of steps counted by the pedometer. Sedentary behavior was analyzed considering different screen devices (television, computer, video game console, tablet computer) and separately the cell phone, and also incorporated sitting time information for weekdays and weekends as obtained by the International Physical Activity Questionnaire. Lastly, information from the food frequency questionnaire was in a form that allowed verification of the number of meals and enabled the use of two-step clustering (TSC), which was important for its interpretation.

According to the review conducted by Leech et al. [10], there are no records of scientific publications using latent class analysis (LCA) or cluster analysis for the assessment of Brazilian adolescents’ lifestyle. Even in the international literature there are few publications analyzing the influence of social and behavioral factors on the lifestyle of female adolescents [13].

The present study, in turn, was an original and pioneering endeavor with latent class analysis with covariates in the assessment of lifestyle from the observation of behaviors adopted by female adolescents in Brazil. When investigating population heterogeneity, a person-centered approach can offer more insight than a variable centered approach. Person-centered techniques aim to group similar individuals based on their characteristics associated, as the manifest variables associated with lifestyle of adolescents. LCA is exploratory in nature and there is no definitive test to facilitate identification of the ‘true’ number of latent classes [44].

Other important consideration observed in the results, and according Laxer et al. [44], there was evidence that health behaviors do not occur in isolation, and that a comprehensive approach that considers the clustering of health behaviors is ideal for promoting health behaviors and reducing chronic disease in youth. Also the study of Hartz et al. [45], our research verified the relationship between MVPA, number of steps and screen time and total time sitting in girls. This finding is consistent with previous findings that increased physical activity level does not necessarily displace sedentary behavior.

Furthermore, the results found will be able to contribute to public health and favor the creation of strategies for practical interventions aimed at helping adolescents embrace a healthier lifestyle. As examples, such interventions might include encouraging the practice of physical activity, lowering of sedentary behavior, compliance with more suitable food frequencies, and early control over alcohol and tobacco use.

## Conclusion

A latent class model with five manifest variables (MVPA, number of steps, ST, sitting time and number of meals) and alcohol consumption as covariate showed itself to be an accurate and objective method for the assessment of female adolescents’ lifestyle. Female adolescents that had ‘never consumed alcohol’ were more likely to belong to class ‘Active & Sedentary lifestyle’ than to class Inactive & Sedentary lifestyle.

Physical activity and sedentary behavior were the manifest variables to influence model formation the most. None of the classes found in the models fitted could identify physically active and non-sedentary adolescents, which strongly suggests these healthy behaviors did not happen together among the students. It was also observed that food frequency was low and alcohol and tobacco consumption were already commonly observed among girls.

Inactivity and sedentarism are coupled to other unhealthy behaviors during adolescence, possibly carrying over into adult life. Thence the necessity to evaluate to what extent lifestyle behaviors may be subject to changes in body composition, risk factors for cardiometabolic diseases and psychosocial problems. Future studies may be able to confirm whether lifestyle as a latent variable is linked to different outcomes related to adolescents’ health.

## Abbreviations

CT: Cell Phone Time
FFQ: Food Frequency Questionnaire
LCA: Latent Class Analysis
MET: Metabolic Equivalent
MVPA: Moderate to Vigorous Physical Activities
NM: Number of Meals
PAL: Physical Activity Level
R24hPAR: 24-h physical activity recall
ST: Screen Time
WHO: World Health Organization

## References

[1] Tsai MC, Strong C, Lin CY (2015). Effects of pubertal timing on deviant behaviors in Taiwan: a longitudinal analysis of 7th- to 12th-grade adolescents. J Adolesc.

[2] Albarello F, Crocetti E, Rubini MI (2018). A longitudinal study on the interplay of personal and social identity in adolescence. J Youth Adolescence.

[3] Skinner AC, Ravanbakht SN, Skelton JA, Perrin EM, Armstrong SC (2018). Prevalence of obesity and severe obesity in US children, 1999–2016. Pediatrics.

[4] Koning M, Hoeskstra T, Jong E, Visscher T, Seidell J, Renders C (2016). Identifying developmental trajectories of body mass index in childhood using latent class growth (mixture) modelling: associations with dietary, sedentary and physical activity behaviors: a longitudinal study. BMC Public Health.

[5] Moreno LA, Gottrand F, Huybrechts I, Ruiz JR, González-Gross M, Dehenauw S (2014). Nutrition and Lifestyle in European Adolescents: The HELENA (Healthy Lifestyle in Europe by Nutrition in Adolescence) Study. American Society for Nutrition. Adv Nut.

[6] Klinker CD, Schipperijn J, Christian H, Kerr J, Ersbøll AK, Troelsen J (2014). Using accelerometers and global positioning system devices to assess gender and age differences in children’s school, transport, leisure and home based physical activity. Int J Behav Nutr Phys Act.

[7] Pearson N, Braithwaite RE, Biddle SJH, van Sluijs EMF, Atkin AJ (2014). Associations between sedentary behaviour and physical activity in children and adolescents: a meta-analysis. Obes Rev.

[8] Saunders TJ, Chaput JP, Tremblay MS (2014). Sedentary Behaviour as an Emerging Risk Factor for Cardiometabolic Diseases in Children and Youth. Can J Diabetes.

[9] Lanza ST, Rhoades BL (2013). Latent class analysis: an alternative perspective on subgroup analysis in prevention and treatment. Prev Sci.

[10] Leech RM, Mcnaughton AS, Timperio A (2014). The clustering of diet, physical activity and sedentary behavior in children and adolescents: a review. Int J Behav Nutr Phys Act.

[11] Flynt A (2016). A survey of popular R packages for cluster analysis. J Educ Behav Stat.

[12] Falbe J, Willett C, Rosner B, Field AE (2017). Body mass index, new modes of TV viewing and active video games. Pediatr Obes.

[13] Balantekin KN, Birch LL, Savage JS. Family, friend, and media factors are associated with patterns of weight-control behavior among adolescent girls. Eat Weight Disord. 2017:1–9. 10.1007/s40519-016-0359-4. 10.1007/s40519-016-0359-4 PMC5601019 28315233

[14] Cochran WG (1977). Sampling Techniques.

[15] Brazilian Association of Survey Companies – (ABEP) (2014). Available from: http://www.abep.org/criterio-brasil. Accessed 14 Mar 2014.

[16] Linzer DA, Lewis JB (2011). poLCA: an R package for Polytomous variable latent class analysis. J Stat Softw.

[17] Corder K, Ekelund U, Steele RM, Wreham NJ, Brage S (2008). Assessment of physical activity in youth. J Appl Physiol.

[18] Tudor-Loocke C, Craig CL, Beets MW, Belton S, Cardon GM, Duncan S (2011). How many steps/day are enough? For children and adolescents. Int J Behav Nutr Phys Act.

[19] Bratteby LE, Sandhagen BO, Fan H, Samuelson G (1997). A 7-day activity diary for assessment of daily energy expenditure validated by the doubly labelled water method in adolescents. Eur J Clin Nutr.

[20] World Health Organization (WHO) (2010). Global recommendations on physical activity for health.

[21] Schofield WN (1985). Predicting basal metabolic rate, new standards and review of previous work. Hum. Nutr Clin Nutr.

[22] Brooks GA, Butte NF, Rand WM, Flatt JP, Caballero B (2004). Chronicle of the Institute of medicine physical activity recommendation: how a physical activity recommendation came to be among dietary recommendations. Am J Clin Nutr.

[23] Miranda VPN, Morais NS, Faria ER, Amorim PRS, Marins JB, Franceschini SCC, Teixeira PCT, Priore SE. Body dissatisfaction, physical activity, and sedentary behavior in female adolescents. Rev Paul Pediatr. 2018;36(4):482-90. 10.1590/1984-0462/;2018;36;4;00005. 10.1590/1984-0462/;2018;36;4;00005 PMC6322812 29791682

[24] American Academy of Pediatrics. Council on Communications and Media (2011). Children, adolescents, obesity, and the media. Pedriatrics.

[25] Guedes DP, Lopes CC, Guedes JERP (2005). Reproducibility and validity of the international physical activity questionnaire in adolescents. Rev Bras Med Esporte.

[26] Jun L, Qingmin L, Yanjun R, Gong T, Shengfeng W, Liming L (2011). Socio-demographic association of multiple modifiable lifestyle risk factors and their clustering in a representative urban population of adults: a cross-sectional study in Hangzhou, China. Int J Behav Nutr Phys Act.

[27] Tenório MCM, Barros MVG, Tassitano RM, Bezerra J, Tenório JL (2010). Atividade física e comportamento sedentário em adolescentes estudantes do ensino médio. Rev Bras Epidemiol.

[28] Lawler M, Heary C, Nixon E (2017). Variations in adolescents’ motivational characteristics across gender and physical activity patterns: a latent class analysis approach. BMC Public Health.

[29] Fitzpatrick SL, Coughlin JW, Appel LJ, Tyson C, Stevens VJ, Jerome GJ (2015). Application of latent class analysis to identify behavioral patterns of response to behavioral lifestyle interventions in overweight and obese adults. Int J Behav Med.

[30] Grest CV, Lee JO, Gilreath T, Unger JB (2018). (2018). Latent class analysis of intimate partner violence perpetration and victimization among Latino emerging adults. J Youth Adolescence.

[31] Ameryoun A, Sanaeinasa H, Saffari M, Koenig HG (2018). Impact of game-based health promotion programs on body mass index in overweight/obese children and adolescents: a systematic review and meta-analysis of randomized controlled trials. Child Obes.

[32] Lee I-M, Schiroma EJ, Lobelo F, Puska P, Blair SN, Katzmarzyk PT (2012). Effect of physical inactivity on major non-communicable diseases worldwide: an analysis of burden of disease and life expectancy. Lancet.

[33] Thorp AA, Owen N, Neuhaus M, Dunstan DW (2011). Sedentary behaviors and subsequent health outcomes in adults a systematic review of longitudinal studies, 1996–2011. Am J Prev Med.

[34] Biddle SJH, Bengoecha EG, Weisner G (2017). Sedentary behaviour and adiposity in youth: a systematic review of reviews and analysis of causality. Int J Behav Nutr Phys Act.

[35] Hallal PC, Andersen LB, Guthold R, Haskell W, Ekelund U (2012). Global physical activity levels: surveillance progress, pitfalls, and prospects. Lancet.

[36] Dumuid D, Olds T, Lewis LK, Martin-Fernández JA, Katzmarzyk PTK, Barreira T (2017). Health-related quality of life and lifestyle behavior clusters in school-aged children from 12 countries. J Pediatr.

[37] CGI.br/NIC.br, Regional Center for Studies for the Development of the Information Society (Cetic.br), Research on Internet Use by Children and Adolescents in Brazil - ICT Kids Online Brazil 2016. Disponible in: https://cetic.br/pesquisa/kids-online/. Accessed 2 Oct 2016.

[38] Philippi ST, Leme ACB (2015). Dietary intake and meal frequency of Brazilian girls attending a school-based randomized controlled trial. Nutr Food Sci.

[39] Verly Junior E, Carvalho AM, Fisberg RM, Marchioni DM (2013). Adherence to the food guide for the Brazilian population. Rev Saude Publica.

[40] Hyanos AF, Watts AW, Loth KA, Pearson CM, Neumark-Stzainer D (2016). Factors predicting an escalation of restrictive eating during adolescence. J Adolesc Health.

[41] Maeder N, King K, Moe-Byrne T, Wright K, Graham H, Petticrew M (2016). A systematic review on the clustering and co-occurrence of multiple risk behaviours. BMC Public Health.

[42] McCambridge J, McAlaney J, Rowe R (2011). Adult consequences of late adolescent alcohol consumption: a systematic review of cohort studies. PLoS Med.

[43] Menezes AHR, Dalmas JC, Scarinci IC, Maciel SM, Cardelli AAM (2014). Fatores associados ao uso regular de cigarros por adolescentes estudantes de escolas públicas de Londrina, Paraná, Brasil. Cad Saúde Pública.

[44] Laxer CRE, Brownson RC, Dubin JA, Cooke M, Chaurasia A, Leatherdale ST (2017). Clustering of risk-related modifiable behaviours and their association with overweight and obesity among a large sample of youth in the COMPASS study. BMC Public Health.

[45] Hartz j YL, Ayers C, Adu-Brimpong J, Rivers J, Ahuja C, Powell-Wiley TM (2018). Clustering of health behaviors and cardiorespiratory fitness among U.S. adolescents. J Adolesc Health.

